# Prevalence of chronic pain in Brazil: A systematic review and meta-analysis^✰^

**DOI:** 10.1016/j.clinsp.2023.100209

**Published:** 2023-05-16

**Authors:** Bruno Vitor Martins Santiago, Ana Beatriz Garcez de Oliveira, Gabriel Machado Romão da Silva, Maxuel de Freitas da Silva, Pedro Ernandes Bergamo, Maud Parise, Nivaldo Ribeiro Villela

**Affiliations:** a Faculdade de Ciências Médicas, Universidade do Estado do Rio de Janeiro (UERJ), Rio de Janeiro, RJ, Brazil; b Faculdade de Medicina, Universidade Federal do Rio de Janeiro (UFRJ), Rio de Janeiro, RJ, Brazil; c Departamento de Especialidades Cirúrgicas, Faculdade de Ciências Médicas, Universidade do Estado do Rio de Janeiro (UERJ), Rio de Janeiro, RJ, Brazil

**Keywords:** Chronic pain, Prevalence, Risk factors, Systematic review, Meta-analysis

## Abstract

**Objective:**

This review synthesized existing studies on the prevalence of chronic pain in Brazil and its associated factors to produce a recent estimation to guide public health politics.

**Methods:**

A search was carried out in the Ovid Medline, Embase, Web of Science, and BVS Regional/Lilacs databases to identify population-based cross-sectional studies from 2005 to 2020, which reported the prevalence of benign chronic pain in Brazil (more than three months). The risk of bias was assessed using design, sample size determination, and random selection as essential issues. Pooled prevalence estimates were calculated for chronic pain in the general and elderly populations. The protocol was registered on Prospero (CRD42021249678).

**Results:**

Of the 682 identified, 15 macheted the authors’ inclusion criteria. Chronic pain prevalence in the general adult population ranged from 23.02% to 41.4% (pooled estimate 35.70%, 95% Cis 30.42 to 41.17) and was described as moderate to intense. It was associated with female sex, old age, lower education, intense professional activity, excessive alcohol consumption, smoking, central obesity, mood disorder, and sedentarism. The Southeastern and Southern regions presented a higher prevalence. The prevalence in the elderly population ranged from 29.3% to 76.2% (pooled estimate 47.32%, 95% Cis 33.73 to 61.11). In addition, this population visited doctors more frequently, had more sleep disorders, and was more dependent on daily living activities. Almost fifty percent of both populations with chronic pain reported pain-induced disability.

**Conclusion:**

Chronic Pain is highly prevalent in Brazil and associated with significant distress, disability, and poorly controlled.

## Highlights

• The prevalence of chronic pain in the adult population (35.70%) and older adults (47.32%).
• Differs from region to region and is associated with heterogeneous risk factors.
• Manifested mainly with moderate or severe intensity and with an elevated rate of disability.

## Introduction

Chronic Pain (CP) is a common, complex, and distressing disorder. According to the International Association for the Study of Pain (IASP), CP is “pain which has persisted beyond normal tissue healing time”, which, in the absence of other factors, is generally taken to be 3 to 6 months or longer.^1^ Although commonly present due to an injury or a disease, chronic pain is no longer considered just a symptom but rather a disease. It is a multidimensional phenomenon that involves physical, psychological, and sociocultural aspects and impacts the individual's health and well-being, health care services, and society.

CP is an underestimated healthcare problem, impacting the quality of life.^2^ It has been highlighted as one of the most prominent causes of disability worldwide by the Global Burden of Disease reviews. The systematic analysis considered global, regional, and national incidence, prevalence, and Year Lived with Disability (YLD) for 354 acute and chronic diseases and injuries in 195 countries between 1990 and 2017. Over the 28 years studied, low back pain, headache disorders, and depressive disorders have prevailed as three of the top four leading diseases/conditions that caused people to live with a disability. The persistence of depressive disorders and low back pain is significant given the former's relation with self-harm and the latter with a potential loss of functional status in the workforce.^3^

Many countries recognize that chronic pain represents a major priority and challenge for their public health and healthcare systems. In this sense, it is essential to know the prevalence of chronic pain in each population to define appropriate strategies.

Worldwide, one in five adults suffers from pain, and 1 in 10 adults is diagnosed with chronic pain each year, according to IASP data.^1^ While pain affects all populations, regardless of age, sex, income, race/ethnicity, or geography, it is not equally distributed globally since its prevalence is associated with social and economic conditions. Factors such as Pain coping, and racial/ethnic, occupational, or cultural differences could partially explain this difference.^4^

Brazil is a continental country with significant regional population variability. Data on the prevalence of chronic pain in the country are poor, especially when analyzing neglected subgroups (such as the elderly population, for example) and records from the 5 regions of the country. Therefore, determining chronic pain prevalence in different regions in Brazil and its associated risk factors is essential to guide public health policies.

The primary aim of this review was to synthesize existing data on the prevalence of chronic pain in the adult Brazilian population, through representative studies of the 5 regions of the country, to produce more accurate national estimates. The secondary aim is to explore the type, intensity, location and characteristics of the pain of the population evaluated by the studies and whether sociodemographic, geographic, and psychosocial characteristics are related to prevalence estimates.

## Material and methods

This systematic review and meta-analysis are reported in accordance with the Preferred Reporting Items for Systematic Reviews and Meta-Analysis PRISMA. The study protocol was submitted to the International Prospective Register of Systematic Reviews PROSPERO (registration number CRD42021249678).

### Search strategy and study selection

The authors searched Ovid MEDLINE, EMBASE, Web of Science, and BVS Regional/Lilacs from 2005 to 09/2020 (confirmed after six months: March 2021). The following medical subject heading (MeSh) and text terms were used (Supplemental Table 1) and run with Endnote software.

### Eligibility criteria

The authors included all full-text articles published since 2005 that determined the prevalence of benign chronic pain, lasting three months or longer, in Brazil. The inclusion criteria used were cross-sectional population-based surveys with an adult population aged over 18 years, with self-report diagnoses, written in English, Portuguese, or Spanish.

Reasons for exclusion included the following: (1) Not report the prevalence of benign chronic pain; (2) Conference papers; (3) Reviews; (4) Data from medical record reviews; (5) Studies from which more than one publication has arisen or (6) Abstracts without full text.

### Study outcome

#### Prevalence of chronic pain

The primary outcome was the prevalence of benign chronic pain in adults. Although the specific interpretation of chronic pain may differ across studies, the authors applied the following definition of chronic pain as the basis for inclusion: pain lasting 3 months or longer.^1^ Studies meeting inclusion criteria were classified according to the type of pain investigated, specifically by organ system and anatomic structure per criteria established by the ACTTION^5^ ‒ American Pain Society Pain Taxonomy: These categories included the following: (1) Widespread musculoskeletal pain, (2) Localized musculoskeletal pain, (3) Low back/spinal pain, (4) Neuropathic pain (eg, neuralgia) and (5) Headache.

#### Sociodemographic, geographic, and psychosocial factors related to chronic pain

The authors explored variation in chronic pain prevalence by demographic, geographic, and psychosocial factors known to be related to risk for chronic conditions. Depending on the available data, these factors included but were not limited to (1) Sociodemographic variables (eg, sex, ethnicity/race, occupation, and education), (2) Geographic region, and (3) Psychological and behavioral health variables (eg, depression, anxiety, obesity, and disability).

### Data extraction and risk of bias

After removing the duplicated articles, five researchers (B.V.M.S.; A.B.G.A.; GMRS, M.F.S.; and P.E.B.) trained by the first and third authors (M.P.; N.R.V) extracted data from each article meeting the inclusion criteria using a data extraction form, which was double coded by either the primary or third author; discrepancies were resolved by consensus.

The authors made a standardized form ‒ with an Excel sheet ‒ to extract meaningful information: study locations, year of article publication and data collection, study designs, number and age of the individuals in each study, the period of chronic pain considered in the studies, the prevalence of benign chronic pain (Table 1).

**Table 1:** Details of selected studies.

First author and publication year	N (Gender, Male/ Female%)	City/ State/ Region	Method	Study period	Age	Period of chronic pain considered	Prevalence of chronic pain (%)	Risk of bias (score)[6]
Sá et al.^17^ (2008)	2.297 (44.6/55.4)	Salvador/ Bahia NE	Domiciliary interview	1999‒2000	40.91±14.73	> 6 months	41.4	Low (1)
Cordeiro et al.^8^ (2008)	2.341 (35.41/64.59)	Buriticupu/Maranhão/ NE	Domiciliary interview	2001	30 (from 16 to 98)	> 3 months	23,02	Low (3)
Moraes Vieira et al.^14^ (2012)	1.597 (33.6/66.4)	São Luiz/ Maranhão/ NE	Domiciliary interview	2009‒2010	39.5 ± 16.6	> 6 months	42.33	Low (1)
Cabral et al.^13^ (2014)	826 (31/69)	São Paulo City/ São Paulo/ SE	Domiciliary interview	2011‒2012	51.4 ± 19.3	> 6 months	42.01	Low (1)
Ferreira et al.^11^ (2016)	2.446 (38.1/61.9)	São Paulo City/ São Paulo/ SE	Telephone interview		≃39.8 ± 18.2	> 3 months	28.09	Low (1)
Pereira et al.^15^ (2017)	5.037 (47‒53)	São Paulo City/ São Paulo/ SE	Domiciliary interview	2005‒2007	≃39.0 ± 13.5	> 6 months	30.99	Low (2)
Souza et al.^20^ (2017)	723 (48/52)	Several/ Several/ N, NE, SE, S	Cell phone interview	2015‒2016	≃37.6 ± 0.81	> 6 months	38.45	Low (1)
Souza et al.^19^ (2019)	560 (27.6/72.4)	Pelotas/ Rio Grande do Sul/ S	Interview at primary care office	2018	48.0 ± 17.2	> 3 months	41.48	Low (2)
Blay et al.^7^ (2007)	6.963 (34/66)	Several/ Rio Grande do Sul/ S	Domiciliary interview	1995‒1996	≃69.9 ± 28.1	> 6 months	76.20	Low (3)
Dellaroza et al.^9^ (2013)	1.271 (40.4/59.6)	São Paulo City/ São Paulo/ SE	Domiciliary interview	2006	69.5 ± 17.7	> 6 months	29.66	Low (2)
Pereira et al.^16^ (2014)	872 (37.7/62.3)	Goiania/ Goiás/ M	Domiciliary interview	2010	≃70.4 ± 6.2	> 6 months	52.75	Low (1)
Santos et al.^18^ (2015)	1.656 (37.5/62.5)	Florianópolis/ Santa Catarina/ S	Domiciliary interview	2009‒2010	≃70,0 ± 5.9	> 6 months	30.01	Low (1)
Lini et al.^12^ (2016)	416 (56.7/43.3)	Passo Fundo/ Rio Grande do Sul/S	Domiciliary interview	2011	69.0 ± 7.6	> 3 months	54.57	Low (1)
Torres et al.^21^ (2018)	383 (29/71)	Belo Horizonte/ Minas Gerais/ SE	Domiciliary interview	2008‒2009	75.6 ± 6.1	> 6 months	30.03	Low (1)
Ferretti et al.^10^ (2019)	385 (32.7/67.3)	Chapecó, Santa Catarina/ S	Domiciliary interview	2016	≃71.3 ± 6.7	> 3 months	58.18	Low (1)

N, Northern; NE, Northeastern; M, Midwest; SE, Southeastern; S, Southern. Age is presented as mean ± SD or means (range).

The factors associated with chronic pain were also extracted from the studies: sex; educational level; occupational activity, alcohol consumption; smoker status; central obesity; mental disorder; time activity; self-perception of health; marital status, and region of the country) and will be discussed in this review.

In addition, two researchers assessed the risk of bias for each study using a score of nine items, adapted from Hoy et al.,^6^ to evaluate the articles and, depending on the score, classified as low risk (score 0‒3), moderate risk (score 4‒6), and high risk (score 7‒9) (Supplemental Table 2). Finally, one other researcher helped in the decision process in case of disagreements between reviewers' judgments.

### Missing data

If authors reported incomplete information (eg, providing the prevalence rate in a figure only), they were contacted by the first author (B.V.M.S) with a request to submit this information. Specifically, the authors asked the authors to provide missing descriptive data (i.e., frequencies) to determine prevalence rates in the adult age group (eg, the total number of adults in the sample, number of adults with pain condition and breakout by sex, when possible). Those without a working email were contacted through Research Gate. A reminder was sent 2 weeks after the first contact in case the authors had not responded. If a response was not obtained, the study was excluded.

### Data analysis and syntheses

The authors used a descriptive statistic (percentage) to summarize the prevalence rate from individual studies. Data analyses were performed using RStudio (version 2.1.4). For all tests, *p* < 0.05 was deemed significant.

Random-effects meta-analyses were used to calculate prevalence estimates owing to the high expected heterogeneity between studies. Prevalence statistics were depicted using the event rate. Ninety-five percent Confidence Intervals (CIs) were calculated using the sample size (n) and standard error. The authors calculated an overall prevalence rate across all pain conditions and prevalence rates stratified by pain condition.

The authors assessed the heterogeneity of prevalence estimates among studies using both the Begg's Test and I^2^ statistics. For the I^2^ index, values of 75% or higher represented high degrees of heterogeneity.

### Subgroup analyses

The summaries were described into two groups: the prevalence of benign chronic pain in the general adult population and the elderly population. In addition, the risk/associated factors related to chronic pain were described.

## Results

### Search results

**Fig. 1:**
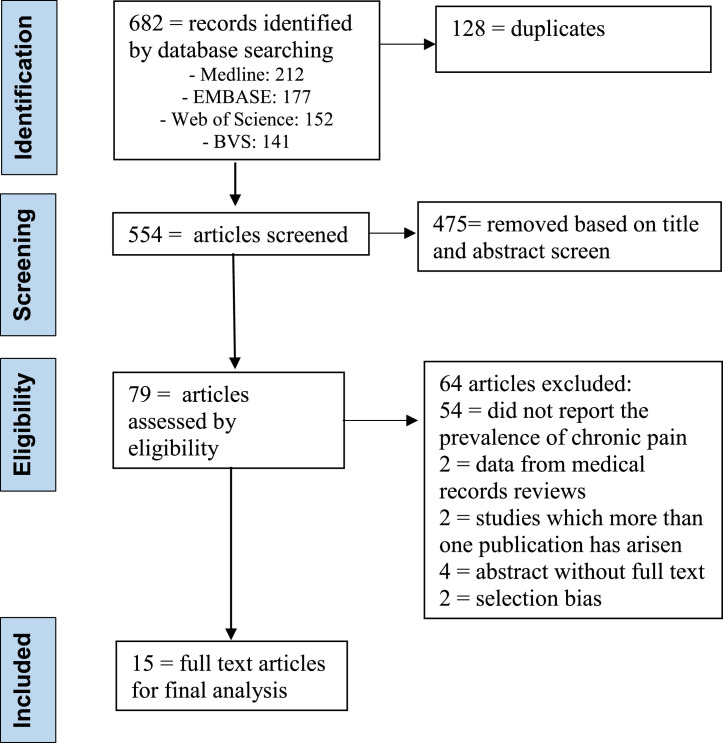
Flow diagram of researched articles.

The authors selected 682 articles for screening, being that 128 were duplicates. Then, after additional screening by title and abstract from the 554 that remained, the researchers excluded 475 articles. Finally, the authors (N.R.V.; and B.V.M.S.;) read the full text of the 79 remaining papers. 64 articles excluded: 54 = did not report the prevalence of chronic pain; 2 = data from medical records reviews; 2 = studies in which more than one publication has arisen; 4 = abstract without full text (authors did not respond) and 2 = selection bias. 15 studies were eligible for final analysis (Table 1).^7^, ^8^, ^9^, ^10^, ^11^, ^12^, ^13^, ^14^, ^15^, ^16^, ^17^, ^18^, ^19^, ^20^, ^21^ The full process and reasons for exclusion can be found in the PRISMA flow diagram (Fig. 1).

#### Characteristics of the included studies

Supplemental Table 2 displays the overall characteristics of the studies included in the meta-analysis. The 15 included studies were published between 2005 and 2020. Sample sizes ranged from 383 to 6.963, including a total of 27.773 participants.

The studies showed wide variations in chronic pain prevalence; therefore, the authors reported the prevalence of the adult and elderly populations.

#### Study quality and risk of bias

Supplemental Table 2 displays the score of risk of study bias, showing how many of the 9 criteria adapted from the Hoy et al.^6^ Quality ratings ranged from 0 to 1. All articles had a low score of bias.

The 15 final studies selected applied different surveys methods: twelve studies performed domiciliar interviews (80%);^7^, ^8^, ^9^, ^10^^,^^12^, ^13^, ^14^, ^15^, ^16^, ^17^, ^18^^,^^21^ one study used a computer-assisted telephone interview;^11^ another accessed the responders by their cell phone using a private database to random the sample,^20^ and one interviewed the users of 38 units of primary care offices.^19^ Ten articles determined the sample size for the chronic pain prevalence study,^10^, ^11^, ^12^, ^13^, ^14^^,^^16^, ^17^, ^18^, ^19^, ^20^ and twelve selected the responders in a random approach.^9^, ^10^, ^11^, ^12^, ^13^, ^14^, ^15^, ^16^, ^17^, ^18^^,^^20^^,^^21^

Different periods for chronic pain were established by studies. As listed in Table 1, pain duration of > 6 months was the most used definition of chronic pain (*n* = 9; 60% of studies), followed by pain lasting > 3 months (*n* = 6; 40% of studies).

Regarding geographic factors related to the prevalence of chronic pain, it should be noted that of the 15 articles that were included in this review, six had participants from the Southern region and eight from the Southeastern region (Table 1).

### Stratified prevalence of chronic pain in adults and elderly population

#### Prevalence of chronic pain in the adult population

**Fig. 2:**
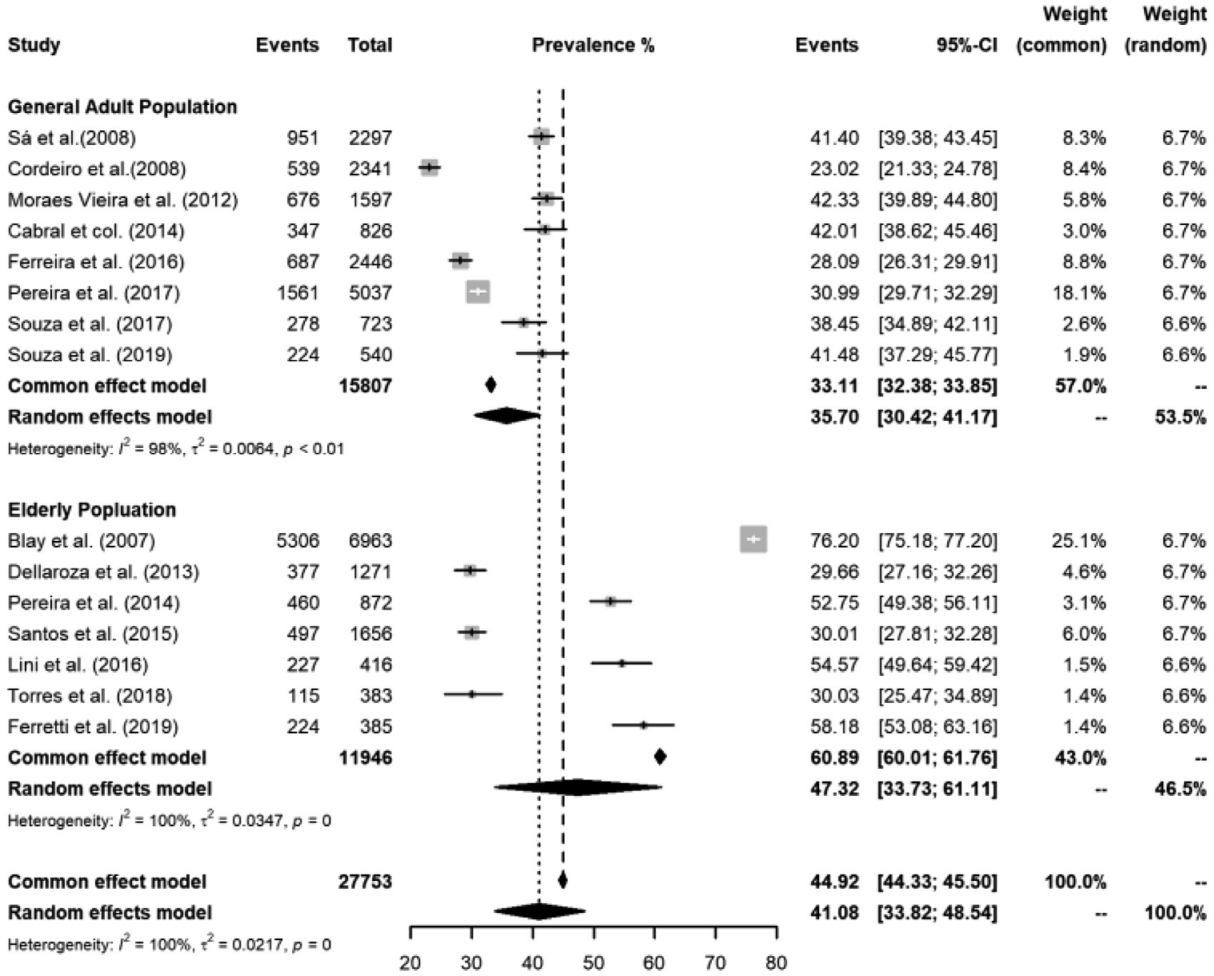
Pooled estimates for chronic pain prevalence in the general adult population by publication date and by subgroups.

Eight studies presented prevalence data of chronic pain in the adult population.^8^^,^^11^^,^^13^, ^14^, ^15^^,^^17^^,^^19^^,^^20^ In addition, five studies evaluated the respondents by a domiciliary interview,^8^^,^^13^, ^14^, ^15^^,^^17^ one by consultation of primary care users,^19^ one by telephone interview,^11^ and one by cell phone interview. The reported prevalence of chronic pain in the adult population ranged from 23.02% to 42.33%, and the overall median prevalence was 35.70% (95% Cis 30.42 to 41.17) I_2_ = 98% / *p* = 0.01 (Fig. 2).

In addition, the articles reported data on the population from different regions of Brazil, and male participants comprised between 27.6% and 48%.

#### Heterogenicity of the studies of the adult population

**Fig. 3:**
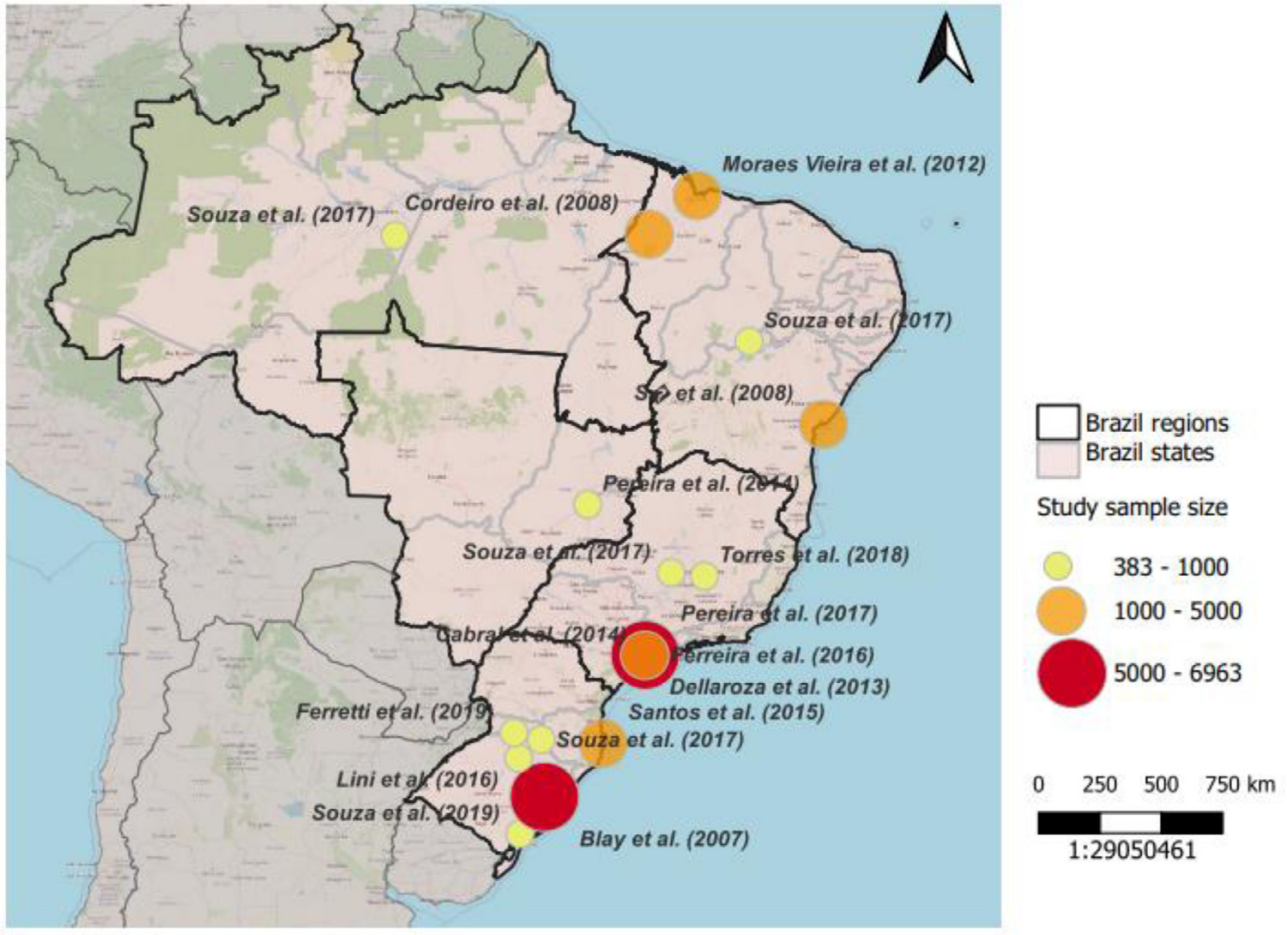
The figure shows the map of Brazil with the identification of the regions where the surveys were carried out and the representation of the sample sizes (author and year of publication).

The studies showed a difference in sex and age distribution that could justify different categories. The difference was also apparent regarding geography (Fig. 3): three studies described the prevalence in São Paulo City,^11^^,^^13^^,^^15^ the biggest city in Brazil; one from diverse regions (Northern, Northeastern, Midwest, Southeastern, and Southern);^20^ three from Northeastern (Maranhão and Bahia),^8^^,^^14^^,^^17^ a more impoverished area;^22^ and one from Southern^19^ (Pelotas) a region with a higher number of elderly^23^ (Table 1).

### Factors associated with chronic pain in the adult population

The studies found an association between chronic pain and gender (female),^8^^,^^11^^,^^13^, ^14^, ^15^^,^^17^^,^^19^^,^^21^ older age,^8^^,^^11^^,^^13^, ^14^, ^15^^,^^17^^,^^19^ lower educational level,^11^^,^^13^^,^^15^ intense or heavy occupational activity,^13^ excessive alcohol consumption for women,^17^ smoking in men and ex-smoker status in both men and women,^17^ presence of central obesity,^17^ anxiety, mood, and mental disorder,^13^^,^^15^ lower laze time activity, and negative self-perception of health.^19^ The Southeastern and Southern regions presented a higher prevalence,^21^ and when respondents indicated their marital status as separated, widowed, divorced, or single, they reported less pain.

### Pain intensity and site in the adult population

The more frequent pain sites were the lumbar region,^8^^,^^13^^,^^17^^,^^19^ cephalic region,^14^ joints,^15^ legs and feet,^11^ and upper limbs.^20^ The most frequent location for the responders with chronic pain with neuropathic characteristics was the lower limbs.^14^ One study found a prevalence of 15% of widespread pain.^20^ In addition, one study reported a prevalence of neuropathic pain of 10%, evaluated by the Douleur Neuropathic 4 Questions (DN4) tools.^14^ Four articles presented the responders’ mean average pain as moderate.^11^^,^^13^, ^14^, ^15^ Another described that 92.4% classified their pain as moderate, intense, strong, or unsupported,^19^ and one study reported pain-induced disability in 52.7% of the responders.^20^

#### Chronic pain prevalence in the elderly

Seven articles presented the prevalence of chronic pain, through domiciliary interviews, in the elderly population.^7^^,^^9^^,^^11^^,^^12^^,^^16^^,^^18^^,^^21^ In addition, they assessed the people from different regions in Brazil: one from São Paulo city,^9^ four from the Southern region,^7^^,^^9^^,^^12^^,^^18^ one from Belo Horizonte (Southeastern),^21^ and another from Goiania (Midwest).^16^ The prevalence of chronic pain ranged from 29.66% to 76.20%, and the overall median prevalence was 47.32% (95% Cis 33.73 to 61.11) I^2^ = 100% / *p* **=** 0.034 (Fig. 2). Male participants comprised between 29% and 56.7% (Table 1).

### Factors associated with chronic pain in the elderly population

The studies found an association between chronic pain in the elderly and gender (female),^7^^,^^10^^,^^12^^,^^16^^,^^18^ lower years of education,^18^ the economic situation,^12^^,^^18^ lower laze time activity, ^18^ sedentarism,^10^^,^^12^ and presence of chronic disease.^10^^,^^16^ In addition, responders with chronic pain visited doctors more frequently in the last 12 months,^21^ had more sleep disorders,^7^ and were more dependent on daily living.^12^

### Pain intensity and pain site in the elderly population

The more frequent pain sites in the elderly responders were lower limbs,^12^^,^^16^ joints,^7^ and the lumbar region.^9^ One study found that 15.1% of the responders reported feeling pain in more than three locations.^16^ In addition, the majority of the individuals with chronic pain described their pain intensity as moderate or severe,^9^^,^^10^^,^^16^^,^^18^ and 48.2% had pain-related disabilities.^21^

## Discussion

This systematic review revealed that the prevalence of benign chronic pain in Brazil is high, worst in the elderly population, differs from region to region and is associated with heterogeneous risk factors. Besides, chronic pain is manifested mainly with moderate or severe intensity and with an elevated rate of disability.

The articles presented variability in the methods and groups studied; therefore, the authors summarized the data from studies that evaluated the prevalence of benign chronic pain in the adult population and the elderly population. Thus, the estimated prevalence of chronic pain in the adult population and older adults is 35.70% and 47.32%, respectively.

Brazil is a continental country with a heterogeneous population and great social inequality. Consequently, gender distribution, domicile location (rural or urban),^24^ access to health care,^25^ and average life expectancy (lower in the north and higher in the south)^26^ vary from region to region or even in the city's distinct neighborhoods.^27^ Thus, determining chronic pain prevalence in Brazil is a great challenge since chronic pain is associated with age, gender, chronic disease, and social condition. Understanding all these regional peculiarities and their impact on health is essential to guide the politics of public health.

Sá et al.,^28^ in a meta-analysis, described a prevalence of chronic pain of 18% in developing countries. However, the presence of a young population, a more significant number of telephone interviews,^29^ a possible regional influence, and other questions related to the methods of the selected articles would justify this low prevalence. On the other hand, Jackson et al.^30^ described wide variability and high prevalence of chronic pain without clear etiology in low and middle-income countries (26%‒42% in the general population and 41%‒81% in the older people), where the elderly and workers had the higher prevalence, which is similar to the findings of the present research.

In a study in the United Kington (UK), Fayaz et al.^31^ described that the prevalence of chronic pain in the general population is 43%. Furthermore, those over 75 years old would be 62% affected. Since the life expectancy is higher in the UK and has an older population, the authors can assume a higher prevalence than in Brazil.

Concerning the pain characteristics, one article found that fifteen percent of the responders had widespread pain,^20^ and in another study, 15.1% of the elderly had pain in more than three locations.^16^ Assuming that central sensitization occurs between five to fifteen percent of the general population,^32^ these data suggest nociplastic pain as a possible diagnostic in these groups. In addition, one article found a prevalence of chronic neuropathic pain of 10%, and the site more affected in this group was lower limbs.^14^

Regarding pain intensity, the responders referred to moderate intense. Additionally, in one study, fifty percent reported pain-related disabilities, and 48.7% referred to their pain treatment as “no effect” or “minor effect”.^20^

Concerning mental health, one article found that responders with pain had 2.3 times more anxiety disorders, 3.3 times more mood disorders, and 2.7 times more mental disorders.^15^ Stubbs et al.^33^ described depression and chronic pain are elevated comorbidities present in low and middle-income countries, independent of anxiety and chronic medical conditions. Furthermore, depression was associated with a higher risk for severe pain.

The articles showed an association between lower economic conditions or lower education, suggesting a relationship between socioeconomic disadvantage and chronic pain.^11^, ^12^, ^13^, ^14^ Besides, central obesity^17^ and sedentarism^10^^,^^12^^,^^18^^,^^19^ were associated with chronic pain. Previous studies support the association of socioeconomic status and chronic pain.^34^ Issues such as inappropriate use of pain coping strategies, race, ethnicity, occupational reasons, exposition to violence, and absence of familiar or social support are some of the involved factors.^3^

The studies with the largest sample representation of participants who responded to the surveys came from the Southern and Southeastern regions. They demonstrated an overall prevalence of chronic pain in adult subjects ranging from 29.66% to 76.20%. These results can be expected, since there is a higher prevalence of elderly people in the population, in addition to a greater number of studies including these regions. In contrast, the smaller sample representation of participants in the Northern and Midwest regions. This can be explained by the smaller number of local studies including; lower human development index and schooling, which could influence access to diagnosis (mainly in the Northern region), corroborating with the findings of Souza et al.^20^

The present review has the merit of getting together the primary studies about Brazil's prevalence of chronic pain and its associated factors. Almost all selected studies performed face-to-face interviews and had a representative number of responders. However, the authors found some limitations. For example, there was wide variability in the study's method; several studies did not describe the prevalence of the general population, being not eligible. Besides, five eligible studies did not calculate the sample size, and the authors did not perform search references from gray literature. Also, interpretation regarding factors associated with chronic pain should be taken into account with caution since the selected articles were transversal studies. The authors decided to summarize studies performed in the last fifteen years since Brazil's population has changed with progressive aging.^23^ Older studies could not reproduce the actual situation of chronic pain prevalence.

## Conclusion

In conclusion, the authors have used the best available articles to demonstrate that benign chronic pain is highly prevalent in Brazil and associated with significant distress, disability, and poorly controlled. Such data suggests the necessity of prioritizing this population's access to qualified and experienced professionals in dealing with chronic pain, improving patient education about its chronic condition, and strengthening the biopsychosocial model, especially in primary care.

## Authors’ contributions

(1) Elaboration of the research question and (2) Literature search: B.V.M.S; M.P and N.R.V; (3) Selection of articles, (4) Data extraction (5) Assessment of methodological quality and (6) Assessment of the quality of evidence: B.V.M.S.; A.B.G.A.; G.M.R.S, M.F.S.; P.E.B.; M.P and N.R.V); (7) Data synthesis (meta-analysis): B.V.M.S and N.R.V; (8) Writing and publishing the results: B.V.M.S; M.P and N.R.V.

## Declaration of Competing Interest

The authors declare no conflicts of interest.

## Appendix Supplementary materials

Image, application 1
