# Supplementary material for: Prevalence of chronic pain in Brazil: A systematic review and meta-analysis
Source: Clinics (Sao Paulo). 2023 May 16;78:100209. doi: 10.1016/j.clinsp.2023.100209 (PMC10206159; doi:10.1016/j.clinsp.2023.100209)
Supplement: Supplementary file 1 [file mmc1.docx]

**CLINICS-D-22-00482_Supplementary Material**

**Supplemental Table 1** Search strategy from different databases.

| **Database** | **Search Strategy** |
| --- | --- |
| Ovid MEDLINE (2005 to 09/2020) | (((Prevalence[mh] OR Prevalence*[tw] OR Prevalent[tw] OR Epidemiology[mh] OR Epidemi*[tw] OR Risk Factors[mh] OR Risk Factor*[tw] OR Associated Factor*[tw] OR Related Factor*[tw] OR Cross-Sectional Studies[mh] OR Cross-Sectional Stud*[tw] OR Cohort Studies[mh] OR Cohort[tw] OR Demography[mh] OR Demograph*[tiab] OR Demographic[tiab] OR Geographic[tiab] OR Sociodemographic[tiab] OR Sociological Factors[mh] OR Sociological*[tiab] OR Social*[tiab] OR Rural[tiab] OR Cities[tiab] OR City[tiab] OR Region*[tiab]) AND (Chronic Pain[mh] OR Chronic Pain*[tw] OR “Chronic Low Back Pain”[tw] OR Widespread Chronic Pain*[tw] OR Persistent Pain*[tw] OR Neuralgia[mh] OR Neuralgia*[tiab] OR Neuropathic Pain*[tiab])) AND (Brazil[mh] OR Brazil*[tw] OR Brasil*[tw])) AND (English[lang] OR Portuguese[lang] OR Spanish[lang]) AND ("2005/01/01"[PDat] : "2020/09/10"[PDat]) |
| EMBASE (2005 to 09/2020) | ('prevalence'/exp OR 'prevalence':ti,ab OR 'prevalence study':ti,ab OR 'epidemiology'/exp OR 'clinical epidemiology':ti,ab OR 'epidemiologic factors':ti,ab OR 'epidemiologic methods':ti,ab OR 'epidemiologic research':ti,ab OR 'epidemiologic research design':ti,ab OR 'epidemiologic studies':ti,ab OR 'epidemiologic study characteristics':ti,ab OR 'epidemiologic study characteristics as topic':ti,ab OR 'epidemiologic survey':ti,ab OR 'epidemiological research':ti,ab OR 'epidemiology':ti,ab OR 'epidemiology model':ti,ab OR epidemi* OR 'risk factor'/exp OR 'relative risk' OR 'risk factor' OR 'risk factors' OR 'related factor*':ti,ab OR 'associated factor*':ti,ab OR 'cross-sectional study'/exp OR 'Cross-Sectional Stud*':ti,ab OR 'cohort analysis'/exp OR 'cohort':ti,ab OR 'demography'/mj OR 'demograph*':ti,ab OR 'geographic':ti,ab OR 'sociodemographic':ti,ab OR 'sociological factors'/mj OR 'sociological*':ti,ab OR 'social*':ti,ab OR 'rural':ti,ab OR 'cities':ti,ab OR 'city':ti,ab OR 'region*':ti,ab) AND ('chronic pain'/exp OR 'chronic intractable pain':ti,ab OR 'pain, chronic':ti,ab OR 'chronic low back pain':ti,ab OR 'widespread chronic pain':ti,ab OR 'persistent pain':ti,ab OR 'chronic pain':ti,ab OR 'neuralgia'/exp OR 'neuralgia' OR 'neuralgia, rheumatic' OR 'neuralgic pain' OR 'neuralgy' OR 'rheumatic neuralgia' OR 'neuropathic pain'/exp OR 'neuropathic pain':ti,ab OR 'pain, neuropathic':ti,ab) AND ('brazil'/exp OR 'brazil':ti,ab OR 'federative republic of brazil':ti,ab OR 'united states of brazil':ti,ab OR 'brazilian'/exp OR 'brazilian':ti,ab OR 'brazilians':ti,ab OR brasil*) AND [embase]/lim NOT ([embase]/lim AND [medline]/lim) AND ([english]/lim OR [portuguese]/lim OR [spanish]/lim) AND [2005-2020]/py |
| Web of Science (2005 to 09/2020) | TS=(Prevalence* OR Prevalent OR Epidemiology OR Epidemi* OR “Risk Factors” OR “Associated Factors” OR “Related Factors” OR “Cross-Sectional Studies” OR “Cross-Sectional Study” OR “Cohort Studies” OR Cohort OR Demography OR Demograph* OR Geographic OR Sociodemographic OR “Sociological Factors” OR Sociological* OR Social* OR Rural OR Cities OR City OR Region*) AND TS=(“Chronic Pain” OR “Chronic Pains” OR “Chronic Low Back Pain” OR “Widespread Chronic Pain” OR “Persistent Pain” OR Neuralgia OR Neuralgia* OR “Neuropathic Pain” OR “Neuropathic Pains”) AND TS=(Brazil OR Brazil* OR Brasil*) AND PY=(2005 OR 2006 OR 2007 OR 2008 OR 2009 OR 2010 OR 2011 OR 2012 OR 2013 OR 2014 OR 2015 OR 2016 OR 2017 OR 2018 OR 2019 OR 2020) AND CU=(Brazil) |
| BVS Regional/Lilacs (2005 to 09/2020) | (tw:(Prevalence* OR Prevalent OR Epidemiology OR Epidemi* OR "Risk Factors" OR "Associated Factors" OR "Related Factors" OR "Cross-Sectional Studies" OR "Cross-Sectional Study" OR "Cohort Studies" OR Cohort OR Demography OR Demograph* OR Geographic OR Sociodemographic OR "Sociological Factors" OR Sociological* OR Social* OR Rural OR Cities OR City OR Region* OR Prevalencia* OR Prevalente OR Epidemiologia OR Epidemi* OR "Fatores de risco" OR "Fatores associados" OR "Fatores relacionados" OR "Estudos transversais" OR "Estudo transversal" OR "Estudos de coorte" OR Coorte OR Demografia OU Demografi* OR Geografic* OR Sociodemografic* OR "Fatores Sociologicos" OR Sociologico* OR Social OR Sociais OR Rural OR Cidade* OR Regiao* OR Regioes OR "Factores de riesgo" OR "Factores asociados" OR "Factores relacionados" OR "Estudios transversales" OR "Estudio transversal" OR "Estudios de cohortes" OR Cohorte OR "factores sociologicos" OR sociologicos* OR sociales OR rurales OR Ciudad* OR Region* OR Regiones)) AND (ti:("Chronic Pain" OR "Chronic Pains" OR "Chronic Low Back Pain" OR "Widespread Chronic Pain" OR "Persistent Pain" OR Neuralgia OR Neuralgia* OR "Neuropathic Pain" OR "Neuropathic Pains" OR "Dor cronica" OR "Dores cronicas" OR "Dor lombar cronica" OR "Dor cronica generalizada" OR "Dor persistente" OR Neuralgia* OR "Dor neuropatica" OR "Dores neuropaticas" OR "Dolor cronico" OR "Dolor lumbar cronico" OR "Dolor cronico generalizado" OR "Dolor persistente" OR "Dolor neuropatico")) AND "Brazil" OR "Brasil" AND (db:("LILACS")) AND (year_cluster:[2005 TO 2020]) AND NOT (ti:(treatment OR tratamento OR tratamiento)) |

**Supplemental Table 2** The score of risk of study bias.

| **1.** Was the study’s target population a close representation of the national population in relation to relevant variables, e.g. age, sex, occupation? | **Yes (LOW RISK):** The study’s target population was a close representation of the national population | 0 |
| --- | --- | --- |
|  | **No (HIGH RISK):** The study’s target population was clearly NOT representative of the national population | 1 |
| **2.** Was the sampling frame a true or close representation of the target population? | **Yes (LOW RISK):** The sampling frame was a true or close representation of the target population | 0 |
|  | **No (HIGH RISK):** The sampling frame was NOT a true or close representation of the target population. | 1 |
| **3.** Was some form of random selection used to select the sample, OR, was a census undertaken? | **Yes (LOW RISK):** A census was undertaken, OR, some form of random selection was used to select the sample (e.g. simple random sampling, stratified random sampling, cluster sampling, systematic sampling). | 0 |
|  | **No (HIGH RISK):** A census was NOT undertaken, AND some form of random selection was NOT used to select the sample. | 1 |
| **4.** Was the likelihood of non-response bias minimal? | **Yes (LOW RISK):** The response rate for the study was ≥75%, OR, an analysis was performed that showed no significant difference in relevant demographic characteristics between responders and non- responders | 0 |
|  | **No (HIGH RISK):** The response rate was <75%, and if any alalusis comparing responders and non-responderes was done, it showed a significant diference in relevant demographic characyeristics between respinders and non-responders | 1 |
| **5.** Were data collected directly from the subjects (as opposed to a proxy)? | **Yes (LOW RISK):** All data were collected directly from the subjects. | 0 |
|  | **No (HIGH RISK):** In some instances, data were collected from a proxy. | 1 |
| **6.** Was an acceptable case definition used in the study | **Yes (LOW RISK):** An acceptable case definition was used. | 0 |
|  | **No (HIGH RISK):** An acceptable case definition was NOT used | 1 |
| **7.** Was the study instrument that measured the parameter of interest (e.g. prevalence of low back pain) shown to have reliability and validity (if necessary)? | **Yes (LOW RISK):** The study instrument had been shown to have reliability and validity (if this was necessary), e.g. test-re- test, piloting, validation in a previous study, etc. | 0 |
|  | **No (HIGH RISK):** The study instrument had NOT been shown to have reliability or validity (if this was necessary). | 1 |
| **8.** Was the same mode of data collection used for all subjects? | **Yes (LOW RISK):** The same mode of data collection was used for all subjects. | 0 |
|  | **No (HIGH RISK):** The same mode of data collection was NOT used for all subjects. | 1 |
| **9.** Were the numerator(s) and denominato r(s) for the parameter of interest appropriate | **Yes (LOW RISK):** The paper presented appropriate numerator(s) AND denominator(s) for the parameter of interest (e.g. the prevalence of low back pain). | 0 |
|  | **No (HIGH RISK):** The paper did present numerator(s) AND denominator(s) for the parameter of interest but one or more of these were inappropriate. | 1 |
| **10.** Summary on the overall risk of study bias | **LOW RISK** | **0‒3** |
|  | **MODERATE RISK** | **4‒6** |
|  | **HIGH RISK** | **7‒9** |

Adapted from Hoy et al.[6]
